# SOFA in the first 24 hours as an outcome predictor of acute liver failure

**DOI:** 10.5935/0103-507X.20180012

**Published:** 2018

**Authors:** Edison Moraes Rodrigues-Filho, Rogério Fernandes, Anderson Garcez

**Affiliations:** 1 Transplant Intensive Care Unit, Hospital Dom Vicente Scherer, Irmandade Santa Casa de Misericórdia of Porto Alegre - Porto Alegre, Rio Grande do Sul (RS), Brazil.; 2 Hepatic Transplant Group, Hospital Dom Vicente Scherer, Irmandade Santa Casa de Misericórdia of Porto Alegre - Porto Alegre (RS), Brazil.; 3 Integrated Network of Institutional Research in Intensive Medicine, Irmandade Santa Casa de Misericórdia of Porto Alegre - Porto Alegre (RS), Brazil.; 4 Postgraduate Program in Collective Health, Universidade do Vale do Rio dos Sinos - São Leopoldo (RS), Brazil.

**Keywords:** Liver failure, Prognosis, Organ dysfunction scores, Liver transplantation

## Abstract

**Objective:**

To describe a cohort of patients with acute liver failure and to analyze the demographic and clinical factors associated with mortality.

**Methods:**

Retrospective cohort study in which all patients admitted for acute liver failure from July 28, 2012, to August 31, 2017, were included. Clinical and demographic data were collected using the Epimed System. The SAPS 3, SOFA, and MELD scores were measured. The odds ratios and 95% confidence intervals were estimated. Receiver operating characteristics curves were obtained for the prognostic scores, along with the Kaplan-Meier survival curve for the score best predicting mortality.

**Results:**

The majority of the 40 patients were female (77.5%), and the most frequent etiology was hepatitis B (n = 13). Only 35% of the patients underwent liver transplantation. The in-hospital mortality rate was 57.5% (95%CI: 41.5 - 73.5). Among the scores investigated, only SOFA remained associated with risk of death (OR = 1.37; 95%CI 1.11 - 1.69; p < 0.001). After SOFA stratification into < 12 and ≥ 12 points, survival was higher in patients with SOFA <12 (log-rank p < 0.001).

**Conclusion:**

SOFA score in the first 24 hours was the best predictor of fatal outcome.

## INTRODUCTION

Acute liver failure is a rare syndrome with high mortality (60 - 90%), which varies according to the etiology and center responsible for patient management.^(1)^ It is defined by the presence of encephalopathy, interval between jaundice and encephalopathy of up to 26 weeks, and coagulopathy (international normalized ratio [INR] ≥ 1.5) in the absence of previous liver disease.^(1)^ Its etiology is the main prognostic determinant; however, age, duration of the interval between jaundice and encephalopathy, INR value, factor V levels, encephalopathy grade, total serum bilirubin levels, and serum creatinine are also important.^(2)^ Liver transplantation may be the only curative alternative for selected patients.^(2)^

In Brazil, there are few studies evaluating the outcomes and associated risk factors in patients with acute liver failure. The scarce reports usually occur in the context of patients with indication for liver transplantation, performed or not. The Model for End-stage Liver Disease (MELD) score, calculated retrospectively with pre-transplant data, was significantly higher in patients undergoing liver transplantation for acute liver failure who did not survive after surgery (n = 8; MELD = 51.86 ± 12.3) than in patients who underwent liver transplantation and survived (n = 9; MELD = 38.47 ± 7.1).^(3)^ Viana et al. evaluated 20 patients with acute liver failure and criteria for liver transplantation, of which 12 were transplanted and 8 were not. Among the transplanted patients, the mean MELD was 36. Seven patients remained alive with good liver function at a mean follow-up of 26.2 months.^(4)^

In Brazil, the Brazilian Transplantation Registry (*Registro Brasileiro de Transplantes* - RBT), managed by the Brazilian Association of Organ Transplantation (Associação Brasileira de Transplantes de Órgãos - ABTO), recorded 1,880 liver transplants performed in 2016. Of these, 150 were performed in Rio Grande do Sul, including both adult and pediatric recipients, but the RBT does not specify diagnoses.^(5)^ Recently, Lauer et al. reported their experience with 250 transplants performed on 236 patients, of which only 2.4% were performed due to acute liver failure.^(6)^

In this study, our objective was to describe a cohort of patients with acute liver failure and to analyze the demographic and clinical factors associated with mortality.

## METHODS

This retrospective and single-center cohort study was conducted in an intensive care unit (ICU) with 11 beds in a tertiary hospital in the South region of Brazil. The medical team consisted of 17 intensivists, with coverage of 2 physicians per shift, every 24 hours, 7 days a week.

The hospital where the study was conducted is a hospital for hematological and solid organ transplant recipients, with a specialized ICU for this purpose. The unit is a reference for transplanted patients in the immediate postoperative period and with late complications and is also a reference for hospitalization of patients with suspected acute liver failure referred by the municipal and state regulatory centers. The indications for transplantation in cases of acute liver failure were determined by the King's College Criteria, according to the technical board of the Central Transplantation Center.

All patients aged ≥ 18 years admitted for acute liver failure in the ICU of the hospital from July 28, 2012, to August 31, 2017, were included in the study. The acute liver failure definition adopted was previously described in the literature.^(1)^ O'Grady's acute liver failure classification was used:^(7)^ hyperacute when the time interval between jaundice and hepatic encephalopathy was zero to 7 days; acute per se if the interval was 8 to 28 days; and subacute if the interval was > 28 days.

Patient data were entered in the Epimed Monitor System site (Epimed Solutions, Rio de Janeiro, Brazil). There were no losses to follow-up. The Simplified Acute Physiology Score (SAPS) 3^(8,9)^ and the Sequential Organ Failure Assessment (SOFA)^(10)^ were measured considering the data collected in the first hour and in the first 24 hours after ICU admission, respectively. The MELD score was measured with the first laboratory results (bilirubin, INR, and creatinine) available after admission.^(11)^

All patients aged ≥ 18 years admitted to the ICU for acute liver failure were included, regardless of whether they had indications for or had undergone liver transplantation. Only the first admission to the ICU was considered for each patient. Patient data were collected prospectively until the hospital outcome.

This study was approved by the Research Ethics Committee of the *Irmandade Santa Casa de Misericórdia of Porto Alegre* (Brazil Platform CAAE number 19687113.8.2002.5335). The need for free and informed consent was waived, as no intervention was performed and no individual data were disclosed.

Statistical data analysis was performed using Stata version 12.0 (StataCorp LP, College Station, Texas, USA). For descriptions of the data from the overall sample, absolute and relative frequencies were used for categorical variables and measures of central tendency and dispersion for continuous numerical variables. A bivariate analysis was performed to test possible associations between mortality and the characteristics investigated in the study (independent variables), using the Pearson chi square test for heterogeneity of proportions (categorical variables) or linear trends (ordinal variables). For comparison between means (continuous numerical variables), Student's *t* test was used. A significance value less than 5% (p < 0.05) was considered statistically significant. Subsequently, the crude odds ratio (OR) was estimated for the associations investigated, including their respective 95% confidence intervals (95%CI). In addition, the areas under the receiver operating characteristic curve (AUROCs) for the prognostic scores were obtained and compared using the chi-square test for equality between AUROCs, using the algorithm suggested by DeLong et al.^(12)^ Kaplan-Meier survival curves were also obtained for the score that best predicted mortality (SOFA), comparing two groups of patients classified according to the best cutoff point identified by the sensitivity and specificity values obtained in the AUROCs. The Cox-Mantel log-rank test was used to compare survival between the two groups.

## RESULTS

A total of 40 patients with acute liver failure were hospitalized over a period of slightly more than 5 years. The mean age of the patients was 44.3 years (± 12.8 years). Table 1 shows the general characteristics of the patient sample. The majority of the patients were female (77.5%) and came from other institutions (72.5%) directly to the study institution's ICU. The most frequent etiology was viral (15; 37.5%). Of the 15 viral cases, 13 were due to hepatitis B virus. There was also one case of acute viral hepatitis A overlapping with chronic viral infection with hepatitis B virus and one case of acute viral hepatitis A with no documented coinfection or chronic liver disease. Nine cases (22.5%) were considered hepatotoxicity: three cases without a defined agent but with pathology of the compatible explanted organ (one of these cases occurred in an HIV-positive patient without antiretroviral treatment); two cases attributed to antiretrovirals in HIV-positive patients; one case attributed to isoniazid; one case attributed to allopurinol; one case of excessive intake of paracetamol in a patient with chronic hepatopathy due to nonalcoholic steatohepatitis (NASH); and one case of intake of kava-kava tea under medical recommendation, a potentially hepatotoxic herbal remedy. Eight cases with undetermined etiology were observed, three of them in HIV-positive patients with no documented coinfection and no hepatotoxic drug intake. Complications in the first 24 hours were frequent, especially the need for mechanical ventilation, in 70% of the cases. According to O'Grady's classification,^(7)^ 57.5% of the patients had hyperacute liver failure, 30% acute liver failure per se, and 12.5% subacute liver failure. Only 35% of the patients underwent liver transplantation. Nineteen patients were listed for liver transplantation, and five of them did not meet King's College Criteria for the procedure. Of these five, one had worsening of INR, and another was characterized as chronic after liver biopsy. In three patients, it was not possible to retrospectively determine the criterion adopted for transplantation listing. Five listed patients were not transplanted: four due to development of refractory shock and one due to brain death prior to organ transplantation. Of the 14 transplanted patients, 7 were discharged from the hospital, and the remaining patients died during hospitalization. The mean time interval between listing and transplantation was 2 days, not considering the patient listed as chronic mainly due to the MELD score, whose time interval was 21 days.

**Table 1:** General characteristics of the sample and the in-hospital mortality distribution

Characteristics	Total sampleN = 40	Hospital outcome		p value	OR (95%CI)
		Discharge N = 17	Death N = 23		
	n (%)	n (%)	n (%)		
Sex				0.176*	
Male	9 (22.5)	2 (22.2)	7 (77.8)		1
Female	31 (77.5)	15 (48.4)	16 (51.6)		0.30 (0.05 - 1.71)
Age range (years)				0.061^†^	
18 - 39	15 (37.5)	9 (60.0)	6 (40.0)		1
40 - 59	19 (47.5)	7 (36.8)	12 (63.2)		2.57 (0.64 - 10.34)
≥ 60	6 (15.0)	1 (16.7)	5 (83.3)		7.50 (0.69 - 81.25)
Origin of admission				0.689*	
External transfer	29 (72.5)	14 (48.3)	15 (51.7)		1
Ward	4 (10.0)	2 (50.0)	2 (50.0)		0.93 (0.12 - 7.55)
Emergency department	4 (10.0)	1 (25.0)	3 (75.0)		2.80 (0.26 - 30.18)
Other	3 (7.5)	0 (0.0)	3 (100.0)		-
Etiology				0.161*	
Viral	15 (37.5)	3 (20.0)	12 (80.0)		1
Drug-related	9 (22.5)	6 (66.7)	3 (33.3)		0.13 (0.02 - 0.82)
Autoimmune	4 (10.0)	1 (25.0)	3 (75.0)		0.75 (0.06 - 10.03)
Pregnancy	4 (10.0)	2 (50.0)	2 (50.0)		0.25 (0.02 - 2.58)
Undetermined	8 (20.0)	5 (62.5)	3 (37.5)		0.15 (0.02 - 1.01)
Complications in the first 24 hours					
Respiratory failure	13 (32.5)	2 (15.4)	11 (84.6)	0.025*	6.88 (1.27 - 37.15)
Mechanical ventilation	28 (70.0)	7 (25.0)	21 (75.0)	0.002*	13.34 (2.63 - 85.68)
Vasopressors	16 (40.0)	2 (12.5)	14 (87.5)	0.005*	11.67 (2.14 - 63.64)
Acute kidney injury	18 (45.0)	4 (22.2)	14 (77.8)	0.023*	5.06 (1.25 - 20.48)
Dialysis	12 (30.0)	3 (33.3)	8 (66.7)	0.445*	1.73 (0.42 - 7.11)
Interval between jaundice and hepatic encephalopathy				0.077*	
≤ 7 days	23 (57.5)	7 (30.4)	16 (69.6)		3.27 (0.88 - 12.13)
> 7 days	17 (42.5)	10 (58.8)	7 (41.2)		1
Glasgow Coma Scale score at admission				0.244*	
< 8	16 (40.0)	5 (31.2)	11 (68.8)		2.20 (0.58 - 8.28)
≥ 8	24 (60.0)	12 (50.0)	12 (50.0)		1
Liver transplantation				0.483*	
No	26 (65.0)	10 (38.5)	16 (61.5)		1.60 (0.43 - 5.94)
Yes	14 (35.0)	7 (50.0)	7 (50.0)		1

OR - odds ratio; 95%CI - 95% confidence interval.

* p values for Pearson's chi-square test for heterogeneity of proportions;

† p value for Pearson's chi-square test for linear trend.

The in-hospital mortality rate was 57.5% (95%CI: 41.5 - 73.5).

Table 2 shows the in-hospital mortality distribution according to laboratory data and prognostic scores in patients with acute liver failure. In this sample, patients who developed complications such as respiratory failure and acute kidney injury or who required mechanical ventilation and vasopressors within the first 24 hours after admission were more likely to die than patients who did not develop these complications (Table 1). Worse laboratory value in the first 24 hours of admission for INR (7.1 ± 5.7) and factor V (24.7 ± 17.9) were also associated with a higher occurrence of mortality, as were higher SOFA (13.5 ± 4.3) and MELD (38.7 ± 12.8) scores (Table 2).

**Table 2:** Means and standard deviations of laboratory characteristics and prognostic scores in the total sample

Characteristics	Total sample N = 40	Hospital outcome		p value*	OR (95%CI)
		Discharge N = 17	Death N = 23		
	Mean ± SD	Mean ± SD	Mean ± SD		
Worse laboratory value in the first 24 hours after admission					
Bilirubin	16.0 ± 8.0	15.5 ± 7.6	16.3 ± 8.4	0.732	1.01 (0.94 - 1.10)
INR	5.2 ± 4.9	2.7 ± 1.4	7.1 ± 5.7	0.003	1.46 (1.04 - 2.04)
Factor V	32.2 ± 22.7	41.8 ± 25.1	24.7 ± 17.9	0.017	1.11 (1.02 - 1.20)
Scores					
SAPS 3	58.5 ± 16.5	52.4 ± 9.8	62.9 ± 19.1	0.044	1.05 (1.00 - 1.11)
SOFA	11.2 ± 4.8	8.2 ± 3.7	13.5 ± 4.3	< 0.001	1.37 (1.11 - 1.69)
MELD	34.2 ± 11.7	28.1 ± 6.5	38.7 ± 12.8	0.003	1.11 (1.02 - 1.20)

SD - standard deviation; OR - odds ratio; 95%CI - 95% confidence interval; INR - International Normalized Ratio; SAPS - Simplified Acute Physiology Score; SOFA - Sequential Organ Failure Assessment; MELD - Model for End-stage Liver Disease.

* p values for Student's t test for comparison of means.

**Figure 1:**
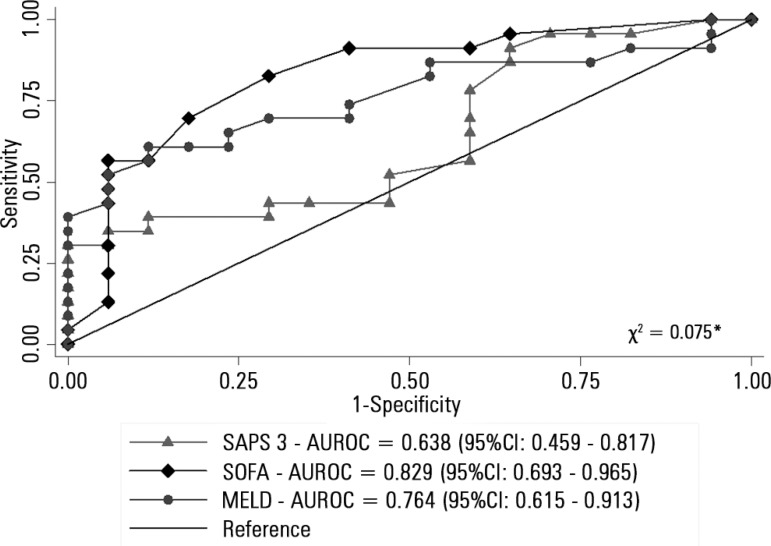
Areas under the receiver operating characteristic curve of prognostic scores for hospital outcome in patients with acute liver failure. (N = 40). SAPS - Simplified Acute Physiology Score; SOFA - Sequential Organ Failure Assessment; MELD - Model for End-Stage Liver Disease; AUROC: area under the receiver operating characteristic curve. * p value for chi-square test of equality between: area under the receiver operating characteristic curve, using an algorithm suggested by DeLong et al.^(12)^

**Figure 2:**
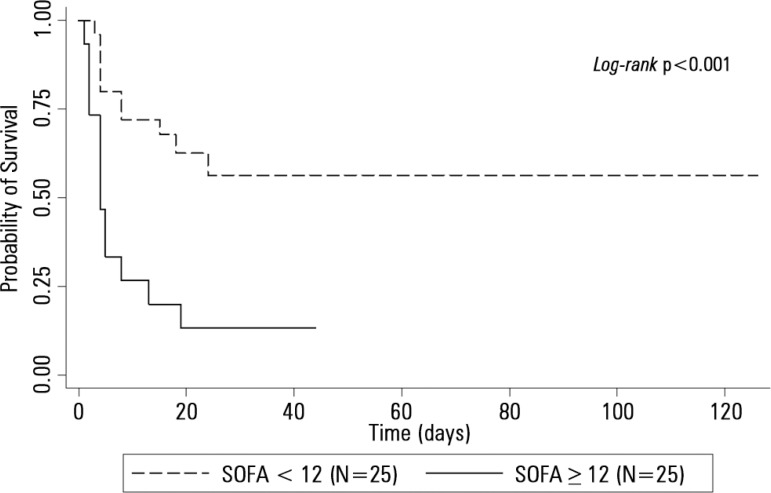
Kaplan-Meier survival curves comparing two groups of patients with acute liver failure classified according to the best cutoff value obtained by the SOFA. SOFA - Sequential Organ Failure Assessment. P-value for Cox-Mantel log-rank test for comparison of survival curves.

Figure 1 compares the AUROCs of the different scores, indicating that the SOFA was a better predictor of mortality. Figure 2 shows the Kaplan-Meier survival curve for the patients, with the SOFA score stratified into < 12 and ≥ 12 points. Survival was lower in patients with SOFA ≥ 12 points (log-rank p < 0.001).

## DISCUSSION

This study describes a large number of patients diagnosed with acute liver failure, allowing better analysis of risk factors for outcomes such as mortality rate. The predominance of females among the patients is compatible with what has been described in European and American studies (73%^(13)^ and 69.3%,^(14)^ respectively). Viral etiology, especially that of hepatitis B virus, was the most common cause in our setting, unlike cohorts in Europe and the United States, where paracetamol intoxication predominated.^(13,14)^ The second most frequent etiology in our setting was hepatotoxic, predominantly due to drugs prescribed for therapeutic purposes. Unlike series reported in Great Britain^(13)^ and the United States,^(14)^ we had only one case attributed to paracetamol. In addition, there were three cases of autoimmune hepatitis and three cases of pregnancy, two of which were due to acute fatty infiltration and one due to extensive hepatic laceration resulting from eclampsia. The seven cases in HIV-positive patients are concerning, considering the prevalence of these patients in the population under antiretroviral treatment or not.^(15)^ The difficulty of transplanting an HIV-positive patient in an emergency situation should be noted because immunological status is often unknown at the time of diagnosis of acute liver disease. The non-use of HIV-positive donors for HIV-positive recipients in Brazil is another factor that limits the supply of organs to these patients in an emergency.^(16)^ Undetermined etiologies accounted for 20% of our cases. In the worldwide literature, indeterminate causes account for less than 15% of cases.^(13)^ This difference likely reflects an inability to recognize hepatotoxic or even viral injuries, along with diagnoses such as autoimmune hepatitis and other less common ones.

Among the complications present in the first 24 hours, the high needs for mechanical ventilation, vasopressor drugs, and dialysis characterize the severity of our population, likely because most patients come from other institutions, many with relatively late recognition of the syndrome. The predominance of hyperacute presentations in 40% of the cases at admission should also be emphasized, as these patients are frequently intubated for airway protection and management of intracranial hypertension because they have higher grades of hepatic encephalopathy.^(17)^ Thus, it is not surprising that only 35% of our patients were transplanted, that in-hospital mortality exceeded 57% and that only the SOFA score had statistical significance for predicting fatal outcome. Although not validated for all diagnostic groups responsible for acute liver failure, the SOFA score presented discrimination and calibration superior to the King's College Criteria and the MELD score for paracetamol poisoning.^(18)^ Interestingly, in our study, the SOFA score in the first 24 hours presented better discrimination for the fatal outcome than the MELD score on admission. It is important to note that in a study conducted by Parkash et al. with 91 patients with liver failure, 30 of them with viral hepatitis B, the MELD score was superior to the King's College Criteria for predicting death, with a mean score of 38 in non-survivors^(19)^ - a value similar to ours. The fact that the MELD score and other variables did not reach statistical significance after adjustment was expected, considering the sample size of only 40 patients because there was no power to perform this analysis, as indicated by the 95% CI range of the OR analysis. Nevertheless, attention is drawn to the trend towards a higher risk of death in patients with hyperacute presentations, which is in disagreement with the data from more recent series and more robust cohorts of patients with acute liver failure.^(2,7)^ This finding may reflect a later recognition of the neurological deterioration of these patients, leading to delayed availability of critical care and the possibility of evaluation for emergency liver transplantation.^(2)^

Although only 35% of the patients were transplanted, these numbers are higher than those of the cohort of Ostapowicz et al.,^(13)^ in which 29% of the patients were transplanted, and of Reuben et al.,^(14)^ in which 23.2% of the patients underwent transplantation.

Our study has several limitations. The retrospective nature of the data analysis, even if collected prospectively, prevents defining how many patients were listed and at what time or how many of those listed were not transplanted and for what reasons. Thus, we chose not to test the King's College Criteria available only at admission, as we often consider the criteria along the course of the patient's evolution. We also do not know the exact grade of hepatic encephalopathy at admission, although the Glasgow Coma Scale can better identify the most serious individuals at admission (ECG ≤ 7) because it is less subject to variability in interpretation than the classically used West-Haven Criteria.^(20)^ In addition, we do not have other evolutionary information, which may be important for considering transplantation.^(11)^ Finally, due to the sample size (less than 100 patients), the multivariate logistic regression analysis (adjusted analysis) was impaired.

## CONCLUSIONS

Acute viral hepatitis B was the major etiology, and the SOFA score in the first 24 hours was the best predictor of fatal outcome.
